# Does a Skills Intervention for Parents Have a Positive Impact on Adolescents’ Anorexia Nervosa Outcome? Answers from a Quasi-Randomised Feasibility Trial of SUCCEAT

**DOI:** 10.3390/ijerph18094656

**Published:** 2021-04-27

**Authors:** Julia Philipp, Claudia Franta, Michael Zeiler, Stefanie Truttmann, Tanja Wittek, Hartmut Imgart, Annika Zanko, Ellen Auer-Welsbach, Dunja Mairhofer, Michaela Mitterer, Clarissa Laczkovics, Gabriele Schöfbeck, Elisabeth Jilka, Wolfgang B. Egermann, Janet Treasure, Andreas F. K. Karwautz, Gudrun Wagner

**Affiliations:** 1 Eating Disorders Unit, Department of Child and Adolescent Psychiatry, Medical University of Vienna, 1090 Vienna, Austria; julia.philipp@meduniwien.ac.at (J.P.); claudiaparfuss@gmx.at (C.F.); michael.zeiler@meduniwien.ac.at (M.Z.); stefanie.truttmann@meduniwien.ac.at (S.T.); tanja.wittek@meduniwien.ac.at (T.W.); dunja.mairhofer@meduniwien.ac.at (D.M.); michaela.mitterer@meduniwien.ac.at (M.M.); clarissa.laczkovics@meduniwien.ac.at (C.L.); gabriele.schoefbeck@meduniwien.ac.at (G.S.); elisabeth@jilka.cc (E.J.); egermann.wolfgang@gmail.com (W.B.E.); andreas.karwautz@meduniwien.ac.at (A.F.K.K.); 2 Parkland Clinic, Clinic for Psychosomatic Medicine and Psychotherapy, 34537 Bad Wildungen, Germany; hartmut.imgart@parkland-klinik.de (H.I.); annika.zanko@parkland-klinik.de (A.Z.); 3 Department for Neurology and child and adolescents Psychiatry, 9020 Klagenfurt am Wörthersee, Austria; ellen.auer-welsbach@kabeg.at; 4 Section of Eating Disorders, Department of Psychological Medicine, Institute of Psychiatry, Psychology & Neuroscience, King’s College London, London WC2R 2LS, UK; janet.treasure@kcl.ac.uk

**Keywords:** anorexia nervosa, children and adolescents, caregivers, parents, workshop, online intervention, skills training, motivational interviewing, eating disorders, atypical anorexia nervosa

## Abstract

Skills trainings for caregivers of patients with anorexia nervosa (AN) have been proven to be effective in improving caregiver skills and reducing caregivers’ psychopathology. The effects on patients, especially adolescents, are largely unknown. The aim of this study was to evaluate the effectiveness of a caregivers’ skills training program (Supporting Carers of Children and Adolescents with Eating Disorders in Austria, SUCCEAT, workshop or online version) on adolescents with AN delivered as workshops (WS) or online (ONL). Outcomes are Body-Mass-Index (BMI) percentile, eating psychopathology (Eating Disorder Examination, EDE), attitudinal and behavioural dimensions of eating disorders (Eating Disorder Inventory-2), motivation to change (AN Stages of Change Questionnaire), emotional and behavioural problems (Youth Self-Report) and quality of life (KINDL). All outcome variables significantly improved across both SUCCEAT groups (WS and ONL) and were sustained at 12-month follow-up. The online and workshop delivery of SUCCEAT were equally effective. Most effect sizes were in the medium-to-high range. Full or partial remission was observed in 72% (WS) and 87% (ONL) of patients. Caregiver skills trainings, either delivered as workshops or online modules, are highly recommended to complement treatment as usual.

## 1. Introduction

Anorexia nervosa (AN) is a severe psychiatric disorder with a prevalence of about 1% [1,2] that is defined by persistent restriction of energy intake, intense fear of gaining weight and disturbance in self-perception [1,3,4]. The pathogenesis of the illness is associated with biological, psychological, and sociocultural factors [4,5]. AN tends to begin in adolescence and is often associated with a tendency to a protracted course and high mortality. Two thirds of patients recover from the illness, whereas one third develops a persisting eating disorder (ED) [4]. Early intervention and specialised treatment can positively affect the outcome [1,3,4]. Caring for patients with AN can have a serious emotional impact on parents, causing severe distress and even significant psychiatric symptoms such as anxiety and depression [6,7,8,9]. As described in the cognitive interpersonal model of maintaining factors for EDs [10,11,12], parental distress might lead to interpersonal problems and unfavourable reactions within the family that may maintain the illness and hinder recovery. Therefore, there is an urgent need to provide parents with specific skills and communication strategies to improve their own mental health in order to better support their children and facilitate their recovery. A high number of studies confirm that interventions for parents based on the cognitive interpersonal model of maintaining factors for EDs [10,11,12], developed by Treasure et al., can reduce parental burden, high expressed emotion (HEE) and distress and also improve caregiver skills in parents [13,14,15,16]. Online interventions based on this model, although far less investigated than workshop-based interventions [17], seem to show similar effectiveness when compared to in person workshops for parents [14,15,18]. The use of novel technologies is highly recommended in the field of EDs [4], especially in the context of the actual and ongoing Coronavirus-Disease-2019 (COVID-19) pandemic [19], when access to interventions might be further impeded due to exit restrictions and further measures of social distancing.

Apart from addressing parental burden, HEE and parental skills, the superordinate aims of such caregiver skills trainings are to promote patients’ outcome and to support their recovery [10,11,12,20]. However, whether caregiver skills trainings based on the same model as described above actually have an impact on patients’ outcome is still largely unknown, especially in the long-term. Previous studies indicate that a parental skills training is equally effective as treatment-as-usual (TAU) [20], individual family work [21], or psychoeducational workshops [22,23] concerning patients’ improvement in Body-Mass-Index (BMI) [20,21], AN symptoms [20,22,24], patients’ anxiety and depression [23], distress [20,22,23] as well as quality of life [20,24]. All of these studies evaluated similar interventions based on the cognitive interpersonal model of maintaining factors for EDs [10,11,12], but mainly focused on adult patients [13,20,21,22,23].

Evidence regarding the impact on adolescent patients is particularly sparse. Hodsoll et al. [25] reported a minor advantage in weight gain in adolescents whose parents participated in such a skills programme at 6-month and 12-month follow-up (FU) compared to TAU. Philipp et al. [15] found some improvements in AN symptoms and BMI in adolescents associated with reductions of parental HEE after participating in a parental skills training. Thus, further evaluating the outcome for adolescent patients is crucial in order to shed light on whether adolescents with AN also benefit from highly specialised caregiver interventions. Therefore, the aim of this study was to examine the impact of the “Supporting Carers of Children and Adolescents with Eating Disorders in Austria” (SUCCEAT) intervention, a parental skills training based on the cognitive interpersonal model of maintaining factors for EDs [10,11,12], delivered via workshop (WS) or online (ONL), on adolescent patients’ outcome. To our knowledge, this is the first study comparing two different delivery forms of the described model addressing patients’ outcome. SUCCEAT was translated into German and adapted for parents of adolescents suffering from AN. The main goals are to decrease parental distress and burden and to increase parental skills and knowledge regarding the illness in order to support them to better help their children towards recovery. SUCCEAT addresses coping strategies for parents, communication strategies including Motivational Interviewing (MI) and strategies to enhance motivation to change in the sufferers. We examined whether adolescent AN patients whose parents participated in a WS vs. an ONL version of the SUCCEAT intervention benefitted in terms of BMI percentile, AN symptoms, motivation to change, behavioural and emotional problems, and quality of life at post-intervention and at 12-month FU. Furthermore, we compared remission rates (full, partial, no remission according to the Diagnostic and Statistical Manual of Mental Disorders, Fifth Edition (DSM-5) diagnostic criteria for AN [3]) between patients whose parents participated in the WS and ONL group at 12-month FU.

## 2. Materials and Methods

### 2.1. Trial Design

This trial is a two-arm parallel group quasi-randomised feasibility study. Caregivers of adolescent patients with AN or atypical AN were equally allocated to the SUCCEAT WS and ONL interventions and the patients were monitored regarding their eating disorder and other psychopathology. Further details of the study design are reported elsewhere [14,15,26]. These analyses are part of a larger study, designed to evaluate differences of the carers’ outcomes participating in the SUCCEAT intervention.

### 2.2. Participants

#### 2.2.1. Eligibility Criteria for Participants

Children and adolescents (aged between 10 and 19 years) suffering from AN (F50.0) or atypical AN (F50.1) [27] and their main caregivers (one parent for each child, who spent most of the time with the child) were eligible to participate in this trial. Further inclusion criteria for children and adolescents were receipt of TAU [28] and being fluent in German. Besides, caregivers required Internet access. Exclusion criteria were severe comorbidities of the children and adolescents (e.g., psychosis) and severe morbidity of the caregivers at baseline.

#### 2.2.2. How Participants Were Identified and Consented

Children and adolescents and their main caregivers underwent psychiatric and psychological assessments to ensure the eligibility criteria. The main caregivers and their children who accepted the invitation to participate in this study gave their written informed consent at the same site where they received TAU.

### 2.3. Interventions

#### 2.3.1. Workshop (WS) Group

The SUCCEAT intervention (WS and ONL) was developed for the caregivers (usually parents) of children and adolescents with AN, to alleviate the caregivers’ distress and burden in order to enable them to support their children with AN [14] and to attain long-term changes for a healing atmosphere in the families through reduced HEE and good communication skills [15,29,30,31,32,33]. MI is used to target self-efficacy, to change behaviour that may maintain ED symptoms [34,35], and to convey skills to empower the caregivers to guide their children in a compassionate way. The intervention is based on the cognitive-interpersonal maintenance model [10,11,12], the transtheoretical model (TTM) of change [36] and the antecedent-behaviour-consequence model (ABC model; [37]). The intervention provides MI skills to the parents in order to improve communication within the family and to enhance the patients’ motivation to change (e.g., handling resistance, motivating change through discrepancy). The WS and ONL versions of SUCCEAT are structured interventions, which involve working with caregivers of about eight children and adolescents over the course of eight weekly sessions. Two healthcare professionals coached the participants of the WS and ONL group [14,15,26]. The 2-h WS sessions were held once in a week at the Medical University of Vienna, Department of Child and Adolescent Psychiatry, where the children and adolescents received TAU too. The caregivers received a manual with detailed information based on a book [30,31,32], the German version of a DVD where best-practice examples of how to communicate with the child are shown [38], and weekly handouts.

#### 2.3.2. Online (ONL) Group

The ONL group started with a personal welcome meeting for the caregivers at the Medical University of Vienna, Department of Child and Adolescent Psychiatry, so that the caregivers could get to know each other and the coaches. The caregivers received the DVD as well [38] and got access to the online platform where they could find the same intervention contents as the WS group: the possibility to download the manual [30,31,32] and eight online modules which were activated on a weekly basis and contained the same content as taught in the WS sessions, slightly adapted for online use [33]. The coaches responded to the caregivers’ questions and provided feedback to their progress once a week via the online platform.

### 2.4. Outcome Measurements

Children and adolescents completed the questionnaires at baseline (T0), post-intervention (after 3 months, at the end of the intervention, T1), and 12-month FU (T2). The interventions and the evaluations took place from November 2014 until April 2018. Sociodemographic and clinical characteristics of the patients (e.g., age, sex, BMI percentile, illness duration, type of current treatment) were collected. For further details see Franta et al. [26].

- The BMI percentiles were obtained from clinical measures of height and weight; additionally, sex- and age-specific percentiles were analysed [39].
- The Eating Disorder Examination (EDE; [40,41]) is a semi-structured interview conducted by clinicians to diagnose EDs and to obtain a picture as accurate as possible of the participant’s eating behaviour and attitudes. It is rated through a global score and four subscales: “restraint” (e.g., avoidance of eating; dietary rules), “eating concerns” (e.g., fear of losing control of eating; guilt about eating), “weight concerns” (e.g., preoccupation with weight; desire to lose weight), and “shape concerns” (e.g., importance of shape; discomfort seeing one’s own body). Internal consistency is good (Cronbach’ s alpha for the subscales: 0.73 to 0.86; for the total score: 0.93). In our sample, reliability was high for both, for children below the age of 14 (Cronbach’s alpha: 0.90) and for adolescents aged 14 and above (Cronbach’s alpha: 0.92). The higher the score the higher is the ED psychopathology.
- The Eating Disorder Inventory-2 (EDI-2; [42]) is a self-report measure to assess ED specific and ED associated psychopathology, including a total scale and 11 subscales (“drive for thinness”, “bulimia”, “body dissatisfaction”, “ineffectiveness”, “perfectionism”, “interpersonal distrust”, “interoceptive awareness”, “maturity fears”, “asceticism”, “impulse regulation”, and “social insecurity”). The EDI-2 was validated for children and adolescents aged 10 and above. Excellent data concerning reliability are available (total score: Cronbach’ s alpha: 0.97). For the purpose of this study, only the total score was used. The higher the score the more subjective ED attitudes and behaviours are reported.
- The Anorexia Nervosa Stages of Change Questionnaire (ANSOCQ; [43,44]) is a self-report questionnaire assessing motivation to change related to their ED symptomatology. The children and adolescents select a statement for each item, which represents their current stage of change (precontemplation, contemplation, preparation, action, maintenance). Internal consistency is excellent (Cronbach’s alpha: 0.90). One study [45] shows good reliability for adolescents older than 12 years. Cronbach’s alpha in our sample was 0.91 for children under the age of 14 and 0.94 for adolescents aged 14 and above. The calculated mean score ranges from 1 to 5 with higher scores indicating a higher motivation to change, or an advanced stage of change, respectively.
- The Youth Self-Report (YSR; [46]) is a self-report measure assessing various behavioural and emotional problems (e.g., “anxious/depressed”, “social problems”, “aggressive behaviour”). The YSR was validated for children and adolescents from age 11. Reliability is high (Cronbach’s alpha ≥ 0.91). We used the total score in this study only, with a higher score indicating a higher level of general psychopathology.
- The Health-Related Quality of Life Questionnaire for Children and Adolescents (KINDL; [47,48]) is a self-report measure assessing the quality of life. The total score comprises the subscales: “physical well-being”, “psychological well-being”, “self-worth”, “family”, “friends”, and “school”. The KINDL was validated for children and adolescents aged 7 years and above. Internal consistency is rather high (Cronbach’s alpha: 0.80). The total score ranges from 0 to 100; a higher score indicating a higher quality of life.

### 2.5. Randomisation

SUCCEAT participants of this study were quasi-randomised [49]. Participants were allocated to the WS and ONL group alternately after we scheduled the start dates of the intervention groups. The number of participants per group slightly varied (median size = 7).

### 2.6. Blinding

Neither the researchers nor the study participants involved in this study were blinded to the assigned intervention.

### 2.7. Statistical Analysis

Data analyses were performed with SPSS Statistics 26.0 (IBM Corp., Armonk, NY, USA). Differences between the SUCCEAT WS and ONL groups regarding sociodemographic and clinical characteristics at baseline were analysed using Chi²-tests and ANOVA. We conducted general linear mixed models to analyse symptom changes and group differences in the outcome variables. Moreover, we calculated baseline to 12-month FU effect sizes in terms of Cohen’s d with values below 0.2 indicating small effect, values between 0.2 and 0.8 indicating medium effect and values above 0.8 indicating large effect.

Furthermore, we analysed the clinical significance of outcomes by calculating remission rates at the 12-month FU assessment. According to the DSM-5 criteria [3], full remission was categorised if no diagnostic criterion for AN was met at the 12-month FU assessment. Partial remission was categorised if the BMI was above the 10th sex- and age-specific percentile but either criterion B (intense fear of gaining weight, becoming fat or behaviour that interferes with weight gain) or criterion C (disturbances in self-perception of weight and shape) was still met. No remission was categorised if the BMI was still below the 10th percentile. We performed a Chi²-test to analyse whether remission rates differed between patients from the SUCCEAT WS and ONL groups.

All analyses were done based on the completer sample (participants who provided data at all assessment time points); missing values due to dropout were not replaced.

## 3. Results

### 3.1. Participant Flow

Main caregivers of 102 adolescent patients with AN took part in the SUCCEAT intervention (WS: *n* = 50; ONL: *n* = 52) (see [14] for details). Patients’ outcome data relevant for this study were available for 98 patients (WS: *n* = 48; ONL: *n* = 50). At post-intervention (3 months after the beginning of the intervention), 92 (94%) patients (WS: *n* = 46; ONL: *n* = 46) provided any outcome data. At the 12-month FU, 70 (71%) patients (WS: *n* = 35; ONL: *n* = 35) provided any outcome data. Patients who did not complete the 12-month FU assessment did not differ significantly from patients who provided data in most outcome variables at baseline (BMI: *p* = 0.268; BMI percentile: *p* = 0.641; EDE total: *p* = 0.793; ANSOCQ: *p* =0.524; YSR total: *p* = 0.084; KINDL total: *p* = 0.455). Patients who dropped out at the 12-month assessment had significantly lower EDI-2 total scores at baseline (mean = 57.61, SD = 40.73) compared to patients who provided data (mean = 74.79, SD = 36.81; *t* = 2.109, *p* = 0.038).

### 3.2. Baseline Data

Baseline sociodemographic and clinical characteristics of the patients for each intervention group (WS, ONL) are shown in Table 1. The characteristics of the carers are reported elsewhere [14]. We tested whether patients allocated to the two groups differed regarding sociodemographic and clinical characteristics as well as regarding the baseline scores of the outcome questionnaires. In general, patients from the SUCCEAT WS group showed similar baseline data compared to the ONL group, indicating that the quasi-randomisation has built two similar groups. The average ED duration was the only variable that was significantly different between the groups with patients from the ONL group having a slightly longer ED duration at the point of inclusion in this study compared to patients from the WS group (*p* = 0.041). We checked whether the ED duration had impact on the study effects; as this was not the case, we did not consider this variable as covariate in the final analyses.

### 3.3. Main Outcomes

When contrasting the SUCCEAT WS group to the ONL group using general linear mixed models (Table 2), we found significant main effects of time for all outcome variables indicating improvement in BMI, psychopathology and quality of life independently of the group. We did not find significant time x group interaction effects for any outcome variable indicating that improvements were similar for the WS and ONL format of SUCCEAT. Baseline to 12-month FU effect sizes in terms of Cohen’s d (including 95% confidence intervals) are provided in Table 3. The effect sizes for BMI, BMI percentile and EDE scales were large in both groups. The effect sizes for self-reported ED psychopathology (EDI-2 score), motivation for change (ANSOCQ score), general psychopathology (YSR score) and quality of life (KINDL score) were in the small-to-medium range. In general, effect sizes tended to be slightly higher in the SUCCEAT ONL group compared to the WS group but as all the confidence intervals overlap, this difference is not statistically significant.

### 3.4. Remission Rates

**Figure 1:**
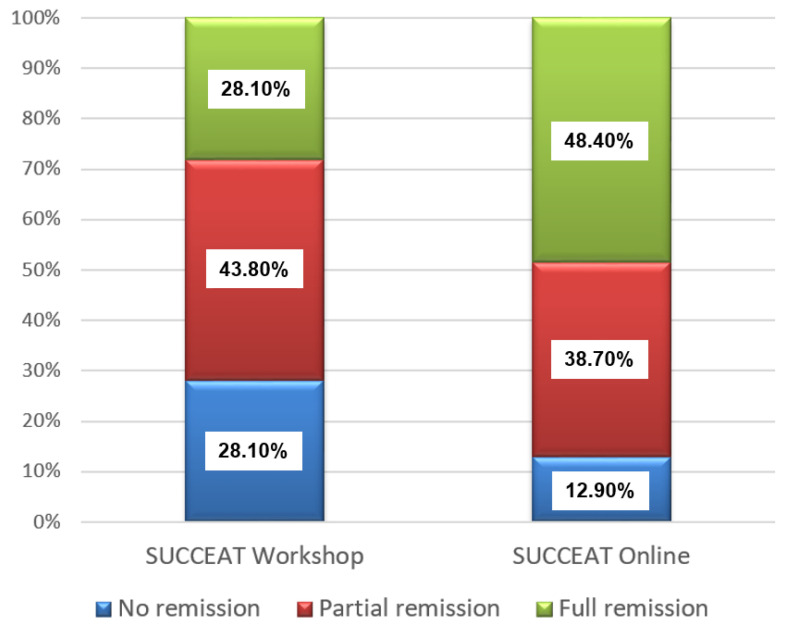
Remission rates at 12-month follow-up for SUCCEAT and comparison group patients.

Figure 1 shows the remission rates of patients of the SUCCEAT WS and ONL group according to the DSM-5 diagnostic criteria at the 12-month FU assessment. Slightly higher rates for full remission were revealed for the SUCCEAT ONL group (48.4% vs. 28.1%), whereas the percentage of patients with no remission was 28.1% in the WS and 12.9% in the ONL group. However, the differences between the SUCCEAT WS and ONL groups were not statistically significant (Chi²(2) = 3.562, *p* = 0.168).

### 3.5. Harms

The caregivers and their children reported no harms. Despite including severely ill patients into the trial, there were no major events reported during the course of the trial and during FU.

## 4. Discussion

The main goal of this study was to examine the impact of SUCCEAT, a skills training for parents based on the cognitive interpersonal model of maintaining factors for EDs [10,11,12], delivered as WS or ONL, on adolescent patients’ outcome. All outcome variables (BMI percentile, ED psychopathology, attitudinal and behavioural dimensions of EDs, motivation to change, emotional and behavioural problems and quality of life) improved in adolescent patients in both groups (WS and ONL) after the intervention. This is similar to other studies, mainly in adults though, that reported improvements in single outcome variables (e.g., BMI [20,21]), AN symptoms [20,22], quality of life [20]). However, our study includes a broad spectrum of different outcome variables that clearly improve over time.

There was no difference with respect to the outcome variables between the WS and ONL format of SUCCEAT across the time points. Patients whose parents participated in the ONL group showed similar improvement as patients with parents participating in the face-to-face WS. We already reported that there were no differences between the WS and the ONL group regarding outcome variables in caregivers [14,15]. The findings of the present study therefore support the conclusion that the SUCCEAT-ONL intervention is as effective as the SUCCEAT-WS intervention in terms of caregivers’ and patients’ outcome. This is important as online interventions have become more and more important in the field of EDs [50,51] and are of high benefit concerning stigmatisation, the availability and the flexibility in time and place [52,53] as they offer an opportunity for families that might otherwise have difficulties accessing further help. Online treatments and online support have gained even more importance during the current COVID-19 pandemic, as their use have strongly been recommended in the field of EDs [19], especially when access to face-to-face treatment might be impeded in case of restrictions. Further research is needed to evaluate online programmes [54], particularly for caregivers. Online interventions might enable patients and their carers to get access to appropriate support earlier, which might be beneficial for the patients’ recovery [55]. On the other hand, online interventions in general and in the field of eating disorders have revealed pitfalls regarding adherence and acceptance, particularly if provided outside of a controlled research setting [56,57]. However, data on the adherence and acceptance of Internet-based interventions or caregivers is scare and needs to be further evaluated when interventions like SUCCEAT are implemented in routine care.

In detail, SUCCEAT delivered via WS and ONL positively affects patients’ outcome regarding weight gain and ED symptoms. Moreover, the positive effects were not limited to ED psychopathology. Motivation to change, general psychopathology and quality of life also improved over the course of the study. The finding that motivation to change increased indicated that patients were more willing to change their problem behaviour after their parents underwent the SUCCEAT interventions. MI and the TTM are key elements of SUCCEAT and aim to increase the intrinsic motivation to change of patients. High motivation to change is related to self-esteem, an active coping style, and predicts remission of AN [45]. Moreover, common comorbidities of EDs, like emotional and behavioural problems as well as anxiety and depression decreased. The quality of life of the adolescent patients increased in terms of emotional, physical and social wellbeing, self-esteem, and everyday functioning in school. This is similar to other studies [24,25], where adolescents reported decreased peer-problems, more pro-social behaviour [25] and adult patients showed an increased quality of life [24]. Sepulveda et al. [22] also reported a marginal improvement of general psychopathology/distress after a similar parental intervention in adult patients though. Yet, our study is the first study of a specialised skills training for parents of adolescent AN patients that revealed a broad spectrum of benefits including motivation to change, general psychopathology and quality of life.

The improvements of all the outcome variables were sustained over time after 12 months in both groups, which is also an important finding, as including a long-term FU is especially rare in this field [13,58,59,60,61]. These findings complement a previous study which has demonstrated that a reduction in HEE in caregivers (after having participated in a skills training) can positively affect the ED psychopathology and BMI in patients [15].

Finally, we investigated the remission rates of the patients at 12-month FU. The percentage of patients with full and partial remission was slightly higher in the WS group compared to the ONL group. About 72% of participants from the WS group showed full or partial remission at 12-month FU, while this percentage was 87% in the ONL group. Accordingly, slightly more patients of the WS group showed no remission at all compared to the ONL group. However, these differences failed to reach significance. Anyway, as it is known that up to 60% of ED patients might recover, whereas about one third of patients is at risk to develop a persisting ED [4], our results somehow reflect similar or even slightly better outcome.

This study has some strengths and limitations. It is the first study focusing on adolescents’ outcome when comparing a WS and an ONL intervention for caregivers including a long-term outcome. The core strengths were the usage of exactly the same design in both SUCCEAT groups (eight sessions or eight modules within the same time frame, the same materials, the possibility to get in touch with other caregivers face-to-face or through a moderated online forum) and access to professional guidance through the same coaches in both groups. Generalisability might however be limited as we included patients with AN and atypical AN and their caregivers only. Future studies should focus on families of patients with bulimia nervosa and binge eating disorder as well. A core limitation was the lack of a randomised control group. More work is needed including a randomised controlled trial and aftercare data to confirm the superiority of SUCCEAT over other specialised family interventions regarding the patients’ outcome. However, SUCCEAT provides the great benefit of an ONL version that is as effective as the WS form. Especially the ONL version might be more cost-effective, more time-saving and may involve more parents at once compared to WS interventions [21].

## 5. Conclusions

This study supports the assumption that adolescent patients with AN might benefit from interventions for their caregivers in addition to TAU, especially in the long-term. Our results strongly support the importance of involving the family in the treatment of adolescents with AN by offering SUCCEAT, a specialised training programme, including psychoeducation, problem solving skills and communication techniques. Equal efficacy of the ONL intervention is a major advantage and an important finding, especially in the current times when online interventions massively gain importance due to the COVID-19 pandemic as they highly increase intervention access. In sum, we recommend integrating a specific skills intervention for parents, potentially as an online support like SUCCEAT-ONL, for caregivers of adolescents with AN into the clinical routine care as an add-on to TAU.

## Figures and Tables

**Table 1:** Clinical baseline characteristics of AN patients.

	SUCCEAT– WS (*N* = 48)	SUCCEAT– ONL (*N* = 50)	*p*
Females (%)	89.6%	96.0%	0.218 ^1^
Age (Mean, SD)	14.65 (1.94)	15.12 (1.80)	0.213 ^2^
ED diagnosis (%)			
AN restrictive	91.7%	90.0%	0.945 ^1^
AN binge/purging	6.3%	8.0%	
Atypical AN	2.1%	2.0%	
ED duration in months (Mean, SD)	10.41 (7.10)	16.03 (16.05)	0.041 ^2^
BMI (Mean, SD)	15.54 (2.12)	16.36 (2.54)	0.087 ^2^
BMI percentile (Mean, SD)	8.67 (13.58)	12.63 (19.77)	0.252 ^2^
Inpatient treatment (%)	47.9%	48.0%	0.993 ^1^
EDE total score (Mean, SD)	3.27 (1.62)	3.32 (1.39)	0.874 ^2^
EDI-2 total score (Mean, SD)	67.32 (39.55)	69.62 (38.79)	0.776 ^2^
ANSOCQ total score (Mean, SD)	3.04 (0.98)	2.81 (1.01)	0.279 ^2^
YSR total score (Mean, SD)	42.39 (22.76)	44.98 (20.65)	0.561 ^2^
KINDL total score (Mean, SD)	56.57 (14.78)	60.16 (13.86)	0.223 ^2^

^1^ Chi² test; ^2^ ANOVA test. Abbreviations: AN, anorexia nervosa; ANSOCQ, Anorexia Nervosa Stages of Change Questionnaire; COMP, comparison group; ED, eating disorder; EDE, Eating Disorder Examination Interview, EDI-2, Eating Disorder Inventory-2; KINDL, Quality of Life questionnaire; ONL, online group; SUCCEAT, Supporting Caregivers of Children and Adolescents with Eating Disorders; WS, workshop group; YSR, Youth Self-Report.

**Table 2:** Results of the general linear mixed models analysing the change in outcome variables by group.

	Mean ± SD			*Time*	*Time x Group*
	Baseline	Post-Intervention	12-Month Follow-Up	*F(df), p*	*F(df), p*
BMI					
Workshop	15.26 ± 2.12	17.04 ± 1.53	18.56 ± 2.61	70.788 (2,122), <0.001	0.321 (2,122), 0.726
Online	16.13 ± 2.31	17.60 ± 2.06	19.04 ± 2.16		
BMI Percentile					
Workshop	7.59 ± 12.39	16.98 ± 16.44	29.41 ± 28.34	37.068 (2,122),	0.073 (2,122), 0.930
Online	10.2 ± 18.23	19.52 ± 20.81	30.64 ± 25.02	<0.001	
EDE total score					
Workshop	3.27 ± 1.46	2.19 ± 1.50	1.68 ± 1.48	54.954 (2,118), <0.001	0.765 (2,118), 0.468
Online	3.53 ± 1.21	2.35 ± 1.52	1.53 ± 1.61		
EDE restraint					
Workshop	3.06 ± 1.89	1.50 ± 1.53	1.12 ± 138	49.061 (2,114), <0.001	0.026 (2,114), 0.974
Online	3.16 ± 1.39	1.59 ± 1.50	1.14 ± 1.53		
EDE eating concerns					
Workshop	2.39 ± 1.60	1.16 ± 1.12	1.21 ± 1.26	33.877 (2,112), <0.001	1.237 (2,112), 0.294
Online	2.59 ± 1.40	1.54 ± 1.43	1.04 ± 1.20		
EDE weight concerns					
Workshop	3.58 ± 1.57	2.41 ± 1.68	1.86 ± 1.78	40.513 (2,118), <0.001	1.096 (2,118), 0.338
Online	3.99 ± 1.49	2.86 ± 1.79	1.72 ± 1.93		
EDE shape concerns					
Workshop	3.71 ± 1.65	3.03 ± 1.99	2.12 ± 1.91	37.742 (2,118), <0.001	0.949 (2,118), 0.390
Online	4.09 ± 1.39	3.01 ± 1.68	1.93 ± 1.79		
EDI-2 total score					
Workshop	64.77 ± 35.14	54.19 ± 36.48	50.49 ± 31.85	6.867 (2,112), 0.002	0.239 (2,112), 0.788
Online	85.25 ± 35.75	69.93 ± 42.65	64.38 ± 51.86		
ANSOCQ total score					
Workshop	3.21 ± 0.78	3.42 ± 1.06	3.65 ± 1.06	9.028 (2,116), <0.001	0.418 (2,116), 0.660
Online	2.79 ± 1.02	3.22 ± 1.18	3.40 ± 1.19		
YSR total score					
Workshop	42.81 ± 23.58	35.28 ± 23.16	38.95 ± 23.93	3.717 (2,118), 0.027	0.211 (2,118), 0.810
Online	51.14 ± 19.11	44.93 ± 20.87	45.25 ± 23.09		
KINDL total score					
Workshop	57.00 ± 15.90	61.84 ± 16.53	67.81 ± 16.09	10.084 (2,118), <0.001	0.912 (2,118), 0.405
Online	56.83 ± 13.90	57.87 ± 14.26	62.79 ± 14.75		

Model specifications: Main effects: group, time, Interaction effects: group x time; Abbreviations: ANSOCQ, Anorexia Nervosa Stages of Change Questionnaire; BMI, Body-Mass-Index; EDE, Eating Disorder Examination Interview, EDI-2, Eating Disorder Inventory-2; KINDL, Quality of Life questionnaire; YSR, Youth Self-Report.

**Table 3:** Baseline to 12-month follow-up effect sizes (Cohens’s d) by group.

	Cohen’s d [95% Confidence Interval]	
	Workshop Group	Online Group
BMI	1.17 [0.72; 1.60]	1.52 [1.01; 2.02]
BMI percentile	0.79 [0.40; 1.17]	1.06 [0.63; 1.49]
EDE total score	1.06 [0.63; 1.49]	1.56 [1.02; 2.08]
EDE restraint	1.11 [0.66; 1.55]	1.22 [0.74; 1.69]
EDE eating concerns	0.76 [0.35; 1.16]	1.37 [0.86; 1.86]
EDE weight concerns	0.92 [0.51; 1.33]	1.29 [0.80; 1.76]
EDE shape concerns	0.88 [0.48; 1.28]	1.40 [0.90; 1.89]
EDI-2 total score	0.26 [−0.11; 0.63]	0.55 [0.17; 0.93]
ANSOCQ total score	0.32 [−0.05; 0.67]	0.63 [0.25; 1.00]
YSR total score	0.12 [−0.23; 0.47]	0.35 [−0.01; 0.72]
KINDL total score	0.54 [0.28; 0.54]	0.36 [−0.01; 1.20]

Abbreviations: ANSOCQ, Anorexia Nervosa Stages of Change Questionnaire; BMI, Body-Mass- Index; EDE, Eating Disorder Examination Interview, EDI-2, Eating Disorder Inventory-2; KINDL, Quality of Life questionnaire; YSR, Youth Self-Report.

## Data Availability

The data that support the findings of this study are available from the corresponding author upon reasonable request.

## Registration

This trial was registered at ClinicalTrials.gov (Identifier: NCT02480907).

## Protocol

The study protocol can be accessed in [26].
